# Hospitalization in neonatal intensive care unit: parental anxiety and satisfaction

**DOI:** 10.11604/pamj.2023.44.55.34344

**Published:** 2023-01-27

**Authors:** Athanasia Voulgaridou, Dimitrios Paliouras, Savas Deftereos, Konstantinos Skarentzos, Evaggelia Tsergoula, Irini Miltsakaki, Panagoula Oikonomou, Maria Aggelidou, Katerina Kambouri

**Affiliations:** 1 University General Hospital of Alexandroupolis, Dragana 1, Alexandroupolis, Greece,; 2 Theageneio Cancer Hospital, Alexandrou Simeonidi 2, Thessaloniki, Greece,; 3 Radiology Department, Democritus University of Thrace, Dragana 1, Alexandroupolis, Greece,; 4 Democritus University of Thrace, Department of Medicine, University General Hospital of Alexandroupolis, Dragana 1, Alexandroupolis, Greece,; 5 Intensive Care Unit, Alexandroupolis University Hospital, Alexandroupolis, Greece,; 6 University General Hospital of Alexandroupolis, Dragana 1, Alexandroupolis, Greece,; 7 Department of Experimental Surgery, Democritus University of Thrace, Dragana 1, Alexandroupolis, Greece,; 8 Department of Pediatric Surgery, Democritus University of Thrace, Dragana 1, Alexandroupolis, Greece

**Keywords:** Greece, Greek, neonatal intensive care unit, parents, quality of care stress, satisfaction

## Abstract

**Introduction:**

the birth of an infant constitutes a challenge for all parents. Stress is intense when an infant is born prematurely or experiences health problems and enters the Neonatal Intensive Care Unit (NICU). Moreover, mothers with premature babies in the NICU would feel frustrated if they are hospitalized in the maternity clinic away from their children. The purpose of this study is to assess the stress experienced by the parents of newborns hospitalized in NICU and its correlation with their level of satisfaction from the services provided during the hospitalization.

**Methods:**

the sample consisted of 102 parents whose children were hospitalized in NICU. Parental Satisfaction questionnaires of NICU and the Picker´s institute were used. Statistical analysis was performed using χ^2^ (chi square) and Pearson´s correlation test (bivariate). STROBE checklist was applied.

**Results:**

out of the 102 parents, 66% were mothers and 33% were fathers. Parents expressed their satisfaction at a rate of 87.8% (±13.9%). In addition, there was strong evidence that the degree of parental satisfaction was significantly related to the age of the mother (p<0.05). The sudden noises from the alarms of the monitoring instruments were strongly correlated with the degree of parents´ satisfaction from the services provided by the NICU (p<0.05). Parents feel less stressful when their child is being monitored (p<0.05).

**Conclusion:**

the results of this study could help the staff of NICU clinics to improve parents´ satisfaction about health services. Proper and adequate communication between parents and health professionals in NICU increases parental satisfaction.

## Introduction

A significant increase in the number of premature births is observed in the last decade. The main causes are assisted reproduction and IVF (in vitro fertilization) due to multiple fetuses in a single pregnancy. Other causes include the increased frequency of induction of preterm birth as a consequence of pathologies such as intrauterine growth retardation, pre-eclampsia and infections [1]. The above reasons lead to an increase in the number of newborns requiring hospitalization, as they have many and severe survival problems.

The Neonatal Intensive Care Units (NICU) consist of health professionals, specialized in the care of premature and full-term newborns. In the last decade, NICU have tripled the survival rate of premature newborns who weigh less than 1000gr. The low birth weight of preterm newborns increases the risk of developing neurodevelopmental disorders in later life, as the tendency to disability is inversely proportional to gestational length (GL) [2]. Based on recent World Health Organization data 15 million preterm newborns are born annually, representing 10-11% of the total number of births in the entire world [3].

Having a premature child or a full-term child with health problems creates intense stress to the parents, disturbing their psychological and social balance [4,5]. The uncertainty about whether the child will survive not only stresses the parents, but also causes problems in the development of the mother-infant relationship [6-8]. The parenting experience, which requires special care, begins in the unfamiliar and unnerving environment of the NICU, which may lead to delayed maternal attachment. According to Shin (2004), mothers experience feelings of ambivalence, shame, guilt and failure associated with social prejudice [9,10]. Mothers believe that their infant in the NICU will have developmental problems and that they blame themselves for the birth of an unhealthy infant.

Mothers with premature babies or with a full-term child with health problems in the NICU would feel frustrated if they are hospitalized in the maternity clinic away from their children. It is common for those mothers to stay close to other mothers who have given birth to healthy babies, while their premature babies are in the NICU [11]. The most frequently reported answers of parents regarding the change of the parental role (their role in caring for their newborns is taken over by the NICU) are the inability to protect the infant from pain, the anxiety, the loss of control, the fear, the uncertainty and the concerns about the outcomes of the premature newborn [10,12]. These findings suggest that the inability to perform a normal parental role is the predominant source of distress.

Patient satisfaction is related to medical services; however, it is not the only factor. In the particular case of neonatal patients who are hospitalized in the NICU and cannot express their own opinion with regard to satisfaction, information is collected indirectly, from their parents. The measurement of parental satisfaction is an important indicator of the quality of services offered by an NICU, as it contributes to the evaluation of the health care provided and its improvement, resulting in the maximization of parental satisfaction with the health care system [13,14]. The purpose of this paper was to assess the stress experienced by the parents of newborns hospitalized in the NICU and the investigation of their satisfaction with regard to the quality of health services provided.

## Methods

**Planning:** the study was conducted in the NICU of a University General Hospital, from 01/11/2018 to 05/05/2019. The board of directors and the scientific ethics committee of the hospital granted their approval for the study, while the education office, the director of the NICU, the supervisor, the doctors and nurses, as well as the parents of the children were informed. The questionnaires were handed off to the parents at the moment of the admission of their newborn to the unit and were collected up to 10 days after discharge. STROBE checklist was applied.

**Sample of the study:** the sample of the study consisted of 102 parents whose newborns were hospitalized in the NICU for a period of at least three days. The sample was selected by random sampling, as long as the necessary conditions were met. Mothers whose newborns were hospitalized in the neonatal unit for less than three days were excluded from the study. Non-probability sampling was used to select the parents to participate in the study. Purposive sampling was specifically preferred. Parents were selected in a way that would serve the purposes of the study.

**Data collection:** two questionnaires were used to conduct the survey. The first one, concerning parental satisfaction was created by Picker Institute Europe called “Picker institute NICU parent satisfaction” [15]. The questionnaire includes 105 closed-ended questions grouped into 10 categories. These categories are: a) before the baby´s birth; b) admission to the neonatal unit; c) transfer to another unit; d) unit staff; e) participation in the care of the baby; f) environment and equipment; g) information and support for parents; h) discharge from the neonatal unit; i) care in another hospital and j) you and your baby. At the end of the questionnaire, the parents rate their overall satisfaction with the quality of the health services provided in the neonatal unit in question, on a scale from 0 to 100. The questionnaire was translated and weighted in the language where the study was performed by two persons who, after personal contact, gave their consent for any use.

The second questionnaire used for parental stress is the Parental Stressor Scale: Neonatal Intensive Care Unit (PSS: NICU) which is a psychometric tool constructed from an adaptation of the Parental Stressor Scale: Neonatal Intensive Care Unit (PSS: NICU) [16]. This questionnaire was written and weighted in the English language, therefore the questionnaire was weighted and translated into the main language of the country the study was performed by one person, with the permission of the questionnaire´s creator.

The particular scale was developed to measure the parents´ perception of stressful situations created by the physical and psychosocial environment of the NICU. The tool consists of 46 questions, divided into 4 categories: 1) images and sounds of the unit (6 questions); 2) appearance and behavior of the newborn (17 questions); 3) variations of the parental role (11 questions) and 4) relationships with the staff of the NICU (11 questions). From the questionnaires distributed to the parents, the subscale on relations with staff was removed, because these statements did not serve a purpose in this study

The questionnaires were sorted, and the results were statistically processed. In the statistical analysis, multivariate statistical analysis was performed using the statistical package IBM SPSS version 24. Quantitative variables were described using figures such as average value, standard deviation and minimum-maximum values. For the description of qualitative variables, the frequencies of occurrence and their respective percentages were used. χ^2^ (chi square) and Pearson´s correlation (bivariate) statistical tests of two variables were performed.

**Ethical approval:** all procedures performed in studies involving human participants were in accordance with the ethical standards of ethical committee of the University General Hospital of Alexandroupolis (Number: 131/1-2-2019) and with the 1964 Helsinki declaration and its later amendments or comparable ethical standards.

## Results

Of the 102 parents who completed the questionnaire, 66% were mothers and 33% were fathers. 94.74% were Greek citizens, while in terms of age, 53.9% were 30-39 and 28.4% were 40-49 years old. In terms of marital status, 96.08% were married, with 50.9% of families having another child, while for 28.4% it was their first one. 45% were graduates of higher education, while 38% were graduates of Middle School or High School. Most of them (44%) were civil servants and 94% had insurance coverage. Those who did not have insurance were unemployed or people engaged in domestic work. Almost 75% resided in a city while the total family income in 37.37% exceeded 1500 euros per month. Most of the mothers exceeded 32 years (73%). Regarding pregnancies, 73% had reached 33 weeks and 42.8% of the newborns had a weight of more than 2500gr. 50% of the newborns required hospitalization for less than 10 days. Finally, the satisfaction of parents reached 87.8% (±13.9%).

Some interesting correlations emerged from the results of our study. There was strong evidence that the degree of parental satisfaction was significantly related to the age of the mother (p<0.05). There is a tendency for a negative linear relationship between the degree of satisfaction and age. This indicates that as the mother´s age increases, a decline in the degree of satisfaction occurs (Table 1).

**Table 1:** satisfaction in correlation with mother´s age

	Degree of satisfaction	Age of mother
Pearson correlation	1	-.345**
Sig. (2-tailed)		.001
N	100	84

** p < .01, two-tailed.

It was observed that the sudden noises from the alarms of the monitoring instruments were strongly correlated with the degree of parents´ satisfaction with the services provided by the NICU (p<0.05) (Table 2). The sudden noises caused an increase in parents´ anxiety, resulting in a lower degree of satisfaction in the corresponding question, in contrast to the continuous noises that did not give a statistically significant difference.

**Table 2:** satisfaction in correlation with the sudden noises from the alarms of the monitoring

	Degree of satisfaction	Sudden noises from the alarms of the monitoring
Pearson Correlation	1	.255*
Sig. (2-tailed)		.016
N	100	89

* p < .05, two-tailed.

The existence of monitors indicates that there is a strong correlation with the degree of parental satisfaction. Therefore, parents feel less stressful when their child is being monitored (p<0.05) (Table 3).

**Table 3:** satisfaction in correlation with the existence of monitoring equipment in NICU

	Degree of satisfaction	The existence of monitoring equipment
Pearson correlation	1	.319**
Sig. (2-tailed)		.002
N	100	95

** p < .01, two-tailed.

The stress caused to parents by not being able to hold their babies when they want to is strongly correlated with their level of satisfaction. Parents who could not hold their baby had a lower level of satisfaction (p=0.05) (Table 4).

**Table 4:** satisfaction in correlation with the ability of parents to hug their children

	Degree of satisfaction	Ability of parents to hug their children
Pearson correlation	1	-.295**
Sig. (2-tailed)		.005
N	100	91

** p < .01, two-tailed.

The anxiety caused by a mother´s fear of holding her baby is strongly correlated with the degree of satisfaction. Mothers who were afraid to hold their baby because it was attached to many machines and were not encouraged to do so, gave a lower degree of satisfaction with the care provided (p<0. 05) (Table 5). Finally, the more the parents felt unable to protect their baby from pain and painful procedures, the lower the degree of satisfaction they gave to the questionnaire (p<0.05) (Table 6).

**Table 5:** satisfaction in correlation with the fear of mother to touch her baby

	Degree of satisfaction	Fear of mother to touch her baby
Pearson correlation	1	-.341**
Sig. (2-tailed)		.004
N	100	69

** p < .01, two-tailed.

**Table 6:** satisfaction in correlation with the feeling of not being able to protect the baby from pain and painful processes

	Degree of satisfaction	The feeling of not being able to protect the baby from pain and painful processes
Pearson correlation	1	-.348**
Sig. (2-tailed)		.001
N	100	85

** p < .01, two-tailed.

It is worth mentioning some interesting correlations of our study, with a marginal statistical result, such as the degree of satisfaction of parents with the baby´s crying for long periods of time (p=0.058 >0.05). The conclusion is that the baby´s crying for long periods of time is not strongly related (the correlation is statistically marginally rejected, the research hypothesis remains) with the degree of satisfaction of parents. Similarly, parents´ degree of satisfaction with bruises and cuts on the baby also influence parents´ scores (p=0.049 <0.05) but it is not the sole criterion as the significance level marginally rejects the research null hypothesis but because the correlations are not strong further investigation is needed.

## Discussion

In the present study, useful conclusions were drawn about the services provided in NICUs, in order to decrease anxiety or uncertainty to parents during the hospitalization of their children. The results of this study could help the staff of NICU clinics to improve parents´ satisfaction about health services.

The age of the mother was shown to influence the degree of satisfaction. As the age of the mother increased, a decrease in the degree of satisfaction was observed. One explanation to this observation of our study may be that mothers with multiple IVFs statistically are older than others so they are distressed and very anxious about their child´s health. These mothers were more demanding and therefore more unsatisfied and most of them have their babies in NICUs. However, there seems to be ambiguous opinions on this issue in literature. In a Californian study measuring mothers´ satisfaction, it was found that older mothers were more satisfied with health care compared to younger mothers [17]. In contrast, a Canadian study showed that mothers´ age and their education level were not significantly associated with satisfaction scores [18]. Recently, Turner *et al*. found that the variable related to advanced parental age, or a very preterm childbirth was associated with higher levels of stress and lower levels of satisfaction, a result that is in line with the present study [19]. In a study by Hagen *et al*. it was shown that the age of parents was positively and significantly associated with their overall satisfaction with the NICU, indicating that older parents were more satisfied [20]. However, Tsironi *et al*. found that younger parents were significantly more satisfied than older parents [21].

It has been reported that, the loud sounds also affect the stress levels of newborns, as their heart rate increases significantly [22]. Loud noises in the NICU that are sudden and uncontrolled lead to anxiety behaviors such as fatigue, overstimulation, fear, hearing impairment, sleep disturbances, as well as body changes, such as increased heart rate, disturbances in arterial and intracranial pressure, fluctuations in blood oxygen saturation, and changes in blood levels of corticosteroid hormones, especially in very preterm infants [23,24]. In the existing study, sudden noises seemed to directly affect the scores given by the survey participants. Unlike continuous noises that are not statistically related to scoring, sudden noises cause parents to consider that the experience is not good, and they give low scores. It would therefore be preferable for staff in NICU clinics to ensure that no sudden noises are generated by various means which are found in the surrounding area, including but not limited to doors, machinery, falling materials or shouting visitors.

The existence of monitoring equipment in this study is a factor strongly related to how satisfied parents feel. It is very important and scientifically interesting that parents value monitoring by medical equipment, such as monitors, very highly, and the satisfaction they expressed was independent of the presence or not of the NICU staff. In contrast, a related study found that the limited opportunity for interaction that parents had during visits appeared to be particularly unpleasant for the majority, while the machines appeared to be a barrier to contact. More generally, the medical technological equipment imposed by the NICU system, contrary to our study, was a barrier to developing physical and emotional contact with the child and was associated with a sense of anxiety in parents [25,26].

Analyzing further the results of the statistical analysis of the present study, the fact that parents feel unable to relieve the pain of their child is a parameter that falls in the same context as the aforementioned cases, resulting in parents not expressing satisfaction from the services provided in the ICU. It is worth mentioning that in some studies, it was found that when mothers were involved in the provision of care, they shifted from a passive to an active role, and did not feel excluded from the care of their newborn. Moreover, when there was facilitation of parents in this matter by the unit staff, they felt more secure, they gained control of the situation, they were more confident and felt more connected to their newborn [9,11,27-29]. Also, a research reported that, although parents experienced a state of alienation, ambivalence and a sense of powerlessness upon separation of their child, ultimately mothers experienced greater responsibility and control when they were involved into the care of their infant [30]. Conversely, fathers were more confident as they left care to healthcare providers trying to balance family life and work. Thus, the encouragement of parents by the staff to participate in some actions allowed for their child in the NICU, would be a way to increase their satisfaction with the services provided.

A study showed that parents with elevated stress levels find the NICU environment even more stressful, particularly in relation to their interactions with staff and their infants´ appearance and behavior in terms of crying, nearby equipment and incisions and surgery [31]. The present study showed similar results, but with marginal results, which proves that perhaps more studies should be performed to support or reject this view. In order to reduce parental anxiety and distress, it is important for staff to focus on communicating with parents and ensuring that ongoing discussions and explanations are made in this regard. Particularly helpful might be interventions that focus on helping parents understand and cope with their infant´s health issues and general stress management techniques.

A group of correlations that emerge from the statistical analysis of this study is the fear of the mother touching the baby, not having time alone with the baby or not being able to hold the baby when she wants to. These are correlated with the score for parental satisfaction and can also lead to useful conclusions and guidelines. In a study by Gale *et al*., it was shown that parental anxiety was reduced by the support offered by the staff, by involving parents in the provision of care for their infant and by providing clear information and open communication with nurses and other health professionals [28]. Conner *et al*., found that parents need care, communication, consistent information, education, monitoring, adequate pain management, involvement in infant care, proximity and support in touching their fragile infant [13]. Phillips *et al*. documented that mothers who did not see or get close to their babies often felt frustrated [32]. Finally, according to Hall, parents should be involved in the care of their sick, fragile newborn in the NICU since parenting is an expression of love [33]. This strengthens the awareness, reduces stress and increases parental satisfaction, which promotes more effective parenting.

Of great importance in this research is the sample examined as well as the geographical location of the clinic where the questionnaires were applied. In particular, the NICU of this University General Hospital serves the health needs of an exceptionally large region. This resulted in difficulty in the distribution and collection of the questionnaires since the parents were not able to visit their child daily due to the long distance. The research could be extended to a larger population and to people with different individual characteristics, also by enriching the questions in order to extract even more interesting and relevant information in some cases.

Equally useful would be the assessment of the experiences of the staff working in NICUs and how they experience their daily interaction with parents and the care of newborns. Finally, the collection of data from numerous and various time periods from the same participants (from the period their child was hospitalized in the NICU to years later) would yield important results not only within the country but also internationally, since the longitudinal studies conducted so far have been limited to collecting data up to a few months after discharge. Some limitations of this study need to be addressed. A limitation is created by the unanswered questions of the questionnaire, for which the researcher is not responsible since the questionnaires were completed voluntarily. Besides, any pressure or even suggestion would destroy the reliability and validity of the research.

Moreover, although the questionnaire was understandable, the completion of the questionnaire by each parent creates the risk of unreliability in their answers as the parent´s judgement is not objective, since they wish to be liked by health professionals, hesitating to state a negative opinion as they feel that the care of their baby is threatened if they express a negative opinion [34].

In the NICU of the aforementioned hospital, a similar study has not been conducted before, hence we could not have a basis of comparison. In the attempt to collect questionnaires in the short time frame of the survey, some of them were completed by both parents together. While this is normally allowed under current guidelines, mother-father answers may create problems in the survey result. Therefore, it would be preferable to repeat the survey with a larger sample of parents, for a longer period of time, in order to have one parent per couple take the questionnaire.

Finally, the choice to complete the questionnaire at home, when the newborn is discharged from the hospital, and the collection of the questionnaire up to 10 days after discharge, affects the objectivity of the parents. This methodology was suggested by Tsironi *et al*. in order to increase the response rate [21].

## Conclusion

The present study shows that parents are very satisfied with the treatment and care provided during their children´s stay in the NICU, but there are some factors that can lead to improvement of the care provided in the NICU. Communication between parents and their baby´s health care providers is essential for the functioning of the NICU. It is important that parents feel that they can communicate with the doctors and nurses in the NICU in order to understand their baby´s condition, participate in the decision-making process and care for their baby appropriately. Stress, technology and the dynamic intensity of the NICU environment can be barriers to communication between parents and health professionals. Adopting a family-centered approach can improve parental satisfaction. Possibly the creation of a forum for parents to provide feedback to improve certain areas, would be an effective practice. Finally, the collaboration of parents and staff in a multidisciplinary approach to improve communication can lead to a significant improvement in parental satisfaction with both communication and quality of care.

### What is known about this topic

- *There are some studies that assess the parent´s stress and satisfaction according to some parameters of the NICU services*.

### What this study adds

- *There is no other study that compare all these parameters together with the stress of the parents;*
- *These results could help for the improvement of NICU services and finally the parental satisfaction*.
